# Radiotherapy Response Assessment of Multiple Myeloma: A Dual-Energy CT Approach With Virtual Non-Calcium Images

**DOI:** 10.3389/fonc.2021.734819

**Published:** 2021-09-23

**Authors:** Philipp Fervers, Erkan Celik, Grischa Bratke, David Maintz, Christian Baues, Simon Ruffing, Philip Pollman-Schweckhorst, Jonathan Kottlors, Simon Lennartz, Nils Große Hokamp

**Affiliations:** ^1^ Department of Radiology, University Hospital of Cologne, Cologne, Germany; ^2^ Department of Radiotherapy and Cyberknife Center, University Hospital of Cologne, Cologne, Germany; ^3^ Chair in Marketing Science and Analytics, University of Cologne, Cologne, Germany

**Keywords:** multiple myeloma, radiation oncology, dual-energy acquisition, computed tomography, virtual noncalcium, irradiation response

## Abstract

**Background:**

Life expectancy of patients with multiple myeloma (MM) has increased over the past decades, underlining the importance of local tumor control and avoidance of dose-dependent side effects of palliative radiotherapy (RT). Virtual noncalcium (VNCa) imaging from dual-energy computed tomography (DECT) has been suggested to estimate cellularity and metabolic activity of lytic bone lesions (LBLs) in MM.

**Objective:**

To explore the feasibility of RT response monitoring with DECT-derived VNCa attenuation measurements in MM.

**Methods:**

Thirty-three patients with 85 LBLs that had been irradiated and 85 paired non-irradiated LBLs from the same patients were included in this retrospective study. Irradiated and non-irradiated LBLs were measured by circular regions of interest (ROIs) on conventional and VNCa images in a total of 216 follow-up measurements (48 before and 168 after RT). Follow-ups were rated as therapy response, stable disease, or local progression according to the MD Anderson criteria. Receiver operating characteristic (ROC) analysis was performed to discriminate irradiated *vs*. non-irradiated and locally progressive *vs*. stable/responsive LBLs using absolute attenuation post-irradiation and percentage attenuation change for patients with pre-irradiation DECT, if available.

**Results:**

Attenuation of LBLs decreased after RT depending on the time that had passed after irradiation [absolute thresholds for identification of irradiated LBLs 30.5–70.0 HU [best area under the curve [AUC] 0.75 (0.59–0.91)] and -77.0 to -22.5 HU [best AUC 0.85 (0.65–1.00)]/-50% and -117% to -167% proportional change of attenuation on conventional and VNCa images, respectively]. VNCa CT was significantly superior for identification of RT effects in LBLs with higher calcium content [best VNCa AUC 0.96 (0.91–1.00), best conventional CT AUC 0.64 (0.45–0.83)]. Thresholds for early identification of local irradiation failure were >20.5 HU on conventional CT [AUC 0.78 (0.68–0.88)] and >-27 HU on VNCa CT [AUC 0.83 (0.70–0.96)].

**Conclusion:**

Therapy response of LBLs after RT can be monitored by VNCa imaging based on regular myeloma scans, which yields potential for optimizing the lesion-specific radiation dose for local tumor control. Decreasing attenuation indicates RT response, while above threshold attenuation of LBLs precedes local irradiation failure.

## Introduction

Multiple myeloma (MM) is one of the most common hematological malignancies and manifests by lytic bone lesions (LBLs) throughout the skeleton in 90% of patients (1). The International Lymphoma Radiation Oncology Group (ILROG) recommends radiation therapy (RT) as palliative treatment of LBLs for local tumor control, pain management, and improvement of functional outcomes (2). In the last decades, the survival of MM patients has continually improved, leading to an increase in palliative RT with growing importance of its dose-dependent side effects (2). In particular, repetitive RT is limited by the patient’s bone marrow (BM) reserve, which is depleted by the myelotoxic effect of ionizing radiation (2). To restrict BM toxicity to the inevitable minimum, recent clinical studies aimed to identify a minimal, individually tailored radiation dose for local tumor control (3, 4). These efforts have been partially adopted by the most recent International Lymphoma Radiation Oncology Group (ILROG) guideline, which suggests radiation doses between 8 Gy in 1 fraction and 30 Gy in 10 fractions for LBLs in MM, depending on the clinical context and therapy goal (2).

Therapy response in MM on a systemic scale has traditionally been monitored by repetitive quantification of the serologic markers (5). However, with advancing capabilities of new imaging modalities, positron emission tomography (PET)/CT has become the gold standard for imaging of lesion-specific treatment response in MM (6). Furthermore, magnetic resonance imaging (MRI) is capable of demonstrating therapy response in MM (7). Established radiological signs of therapy response of an LBL include decreasing tracer uptake in PET/CT, increasing fat fraction as well as decreasing diffusion restriction in MRI, and sclerotic fill-in on conventional computed tomography (CT) images (2, 8, 9).

Attenuation measurement for assessment of a lesion’s therapy response on conventional CT adds up three compartments, which constitute its total density in Hounsfield Units (HU): fat, soft tissue, and bone mineral (10). Assessing either of these compartments in isolation is hampered by the inherent technical limitations of conventional CT, as it does not allow for a decomposition of total attenuation into material-specific fractions. Specifically, attenuation measurements of infiltrated BM in conventional CT inevitably imply the soft-tissue and fatty portion of BM, as well as remaining calcification (5, 11).

Dual-energy CT allows for mitigating this fundamental limitation of conventional CT by simultaneously acquiring two datasets at different energy levels. This allows for material decomposition and thereby virtual subtraction of certain materials of interest from the DECT images, depending on their atomic number (5, 12). By obtaining virtual noncalcium (VNCa) images, DECT enables virtual removal of the bone mineral compartment and provides BM images with similar capabilities to MRI and PET/CT in the context of MM (5, 10, 11, 13). Besides, whole-body low-dose CT is recommended by the IMWG and regularly performed for staging of MM bone disease in clinical practice due to its reasonably low radiation exposure, limited contraindications, and good patient acceptance (14). It would be convenient if DECT acquisitions within this scope could allow for a more accurate monitoring of treatment response.

Hence, the objective of our study was to explore the feasibility of DECT VNCa imaging to assess response to RT in MM.

## Materials and Methods

All procedures performed in studies involving human participants were conducted in accordance with the ethical standards of the institutional and national research committee and with the 1964 Helsinki declaration and its later amendments or comparable ethical standards. Informed consent was waived due to retrospective study characteristics. The study was approved by the institutional review board (ethics committee of the Medical Faculty of the University of Cologne, approval number 20-1480). All imaging procedures were performed for clinical indications. No scan was conducted explicitly for the purpose of this study.

### Patient Enrollment

Study inclusion was based on screening of the picture archiving and communication systems (PACS) and electronic medical records.

Inclusion criteria to our study comprised

1. whole-body low-dose DECT according to the international myeloma working group (IMWG) specified imaging protocol between April 2016 and July 2020,
2. diagnosis of MM according to IMWG criteria,
3. history of RT of at least one LBL,
4. age ≥18 years.

Exclusion criterion was

1. absence of non-irradiated lesions (n=4).

### Definition of Target Lesions

Radiation protocols were reviewed by an MD specialized in radiotherapy. Irradiated LBLs were included according to the RT target volumes.

Exclusion criteria of target lesions were

**Figure 1:**
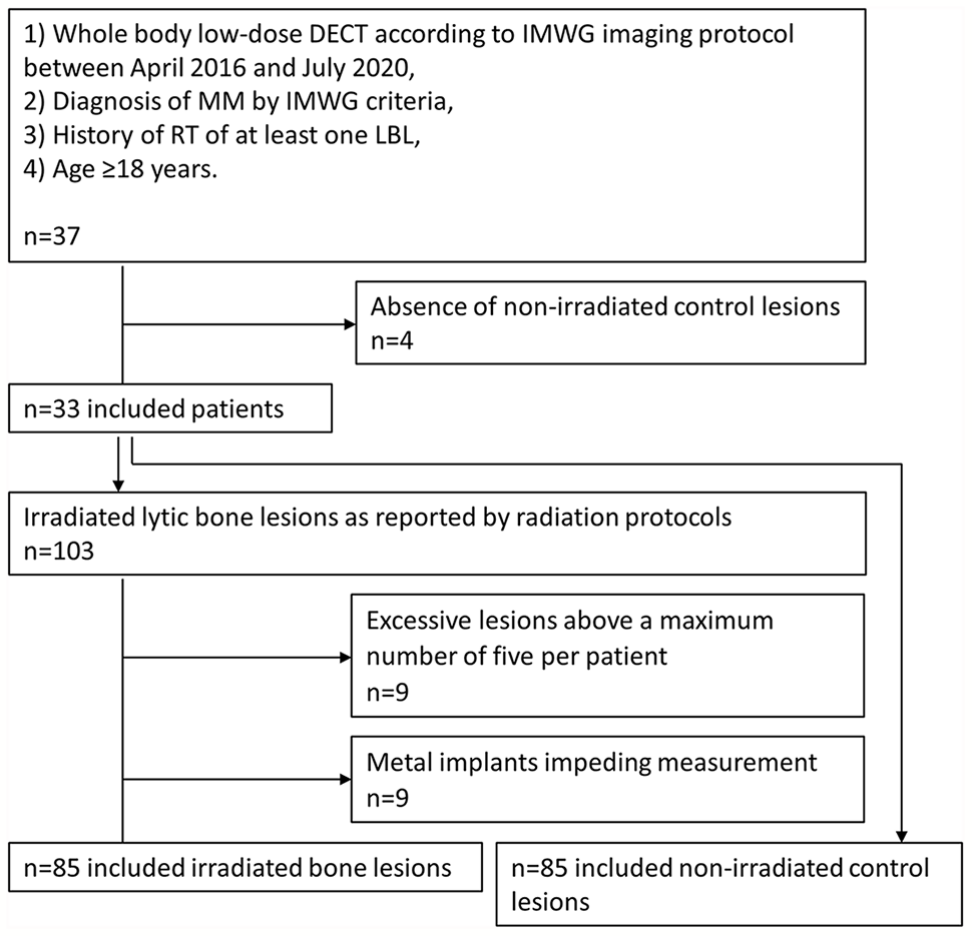
Inclusion chart of patients and target lesions.

1) excessive target LBLs above a maximum number of five per patient (n=9 LBLs),
2) metal implants impeding ROI measurement (n=9 LBLs),

An inclusion chart of patients and target lesions is provided in **Figure 1**.

RT fractionation schedule and total radiation dose were noted for each target lesion. For each irradiated lesion, one representative non-irradiated lesion was defined in the same patient as follows: a certified specialist of musculoskeletal radiology with 7 years of experience analyzed all included CT scans and marked osteolytic lesions with an arrow, while being blinded to clinical patient data and radiation protocols. After unblinding to radiation protocols, control lesions were randomly chosen from the non-irradiated lesions. Each control lesion was evaluated in consensus with another experienced radiologist with 4 years of experience, which did not alter the random selection.

### Assessment of Clinical Data

Clinical records were reviewed by an MD to build the patient population. History of MM specific therapy was noted.

Each included CT scan was assigned to one of two groups of disease activity at the timepoint of imaging (+/- 30 days). The classification of systemic disease activity was achieved by reducing the most recent IMWG uniform response criteria for MM to a binary scale:

1. complete or partial response and stable disease,
2. progressive disease or relapse.

Besides disease progression in imaging, the IMWG criteria assess further systemic criteria, such as rising M-protein levels, increasing infiltration in bone marrow biopsy, or newly developed hypercalcemia, which is described in detail elsewhere (15, 16).

### DECT Imaging

All scans were performed on a commercially available spectral detector DECT scanner (IQon, Philips, Best, The Netherlands), respecting the most recent recommendations for imaging of MM of the IMWG (14). Patients were placed in a head-first supine position. As recommended, no contrast agent was administered. Scan parameters were as follows: Tube current 70 mAs; collimation 32 × 0.625 mm; pitch 0.908; tube voltage 120 kV; computed tomography dose index (CTDIvol) 7.4 mGy. The mean dose length product (DLP) was 1010.7 ± 164.8 mGy*cm.

### Reconstruction of DECT Data

All images were reconstructed in a 512 x 512 matrix. Slice thickness was 2 mm with an overlap of 1 mm. Spectral-based images were reconstructed using a dedicated iterative reconstruction algorithm (17). VNCa images were obtained using the vendor’s proprietary software simulating each voxel’s attenuation without the calcium-specific contribution (IntelliSpace Portal, Spectral Diagnostics Suite, Philips Healthcare). In our study, calcium suppressed images were calculated with a high suppression index (index 25), as suggested in previous studies (13). Detailed information on technical backgrounds of VNCa imaging has been provided before (10).

### Assessment of Conventional and VNCa DECT Images

A representative, circular region of interest (ROI) was placed inside each irradiated and non-irradiated lesion on conventional axial CT slices, carefully excluding the lesion’s borders. The ROI was automatically copied to the VNCa images without specific user interaction. Attenuation in Hounsfield units (HU) on conventional and VNCa images was noted. The reading was performed by a radiologist with 4 years of experience who was blinded to RT history. Adjustment of windowing settings was allowed at all times.

If a prior exam was available, tumor response of irradiated LBLs on conventional CT images was rated by the MD Anderson (MDA) criteria, as adopted by the most recent Response Evaluation Criteria in Solid Tumors (RECIST) (18). Therapy response of each irradiated lesion was assessed by comparison to the immediate previous examination. The MDA criteria comprise a scale of four response categories:

1) complete response with a new complete sclerotic fill-in,
2) partial response with a new partial sclerotic fill-in or development of a sclerotic rim,
3) progressive disease with ≥25% increase in lesion size,
4) stable disease, conforming to neither of the categories above (18).

Both the Hounsfield unit (HU) measurements and the MDA ratings were repeated for a random subset of 30% of the data by two additional readers with 3 and 5 years of experience (128 ROI measurements and ratings each, respectively) to assess inter-reader variability.

### Analysis of VNCa and Conventional Attenuation in Lytic Bone Lesions With and Without Irradiation

The absolute attenuation of irradiated LBLs in HU and the corresponding non-irradiated lesions were noted for each examination. If an examination before RT was available, the proportional change of lesion attenuation before RT *vs*. after RT in percent (%) was calculated. For comparison of the performance of VNCa and conventional CT images, receiver operating characteristic (ROC) analysis was performed with the predictors “percentage attenuation change on conventional/VNCa CT” (if a pre-irradiation study was available), “absolute post-irradiation attenuation on conventional/VNCa CT” (if no pre-irradiation study was available), and the binary outcome “irradiated lesion *vs*. non-irradiated lesion”.

The bone mineral portion of attenuation of the LBLs was estimated by the difference of their attenuation on conventional CT (*att*_conventional_) and VNCa (*att*_VNCa_) images. This method was based on the assumption that *att*_conventional_ adds up all three compartments of bone attenuation (fat, soft-tissue, bone mineral), while *att*_VNCa_ solely consists of the fat and soft-tissue compartments, attributing the discrepancy between both to the bone mineral compartment (10).

### Subset Analysis for Evaluation of Irradiation Effects

Two subsets of particular interest were defined for individual analysis:

1) LBLs with high calcium content, which carry the highest potential to benefit from virtual calcium suppression. To elaborate on this possible benefit, ROC analysis was performed for lesions grouped by their calcium content (above the 25^th^, 50^th^, 75^th^, and 90^th^ percentiles) with the predictors “absolute attenuation on conventional/VNCa CT” and the binary outcome “irradiated *vs*. non-irradiated lesions.”
2) Locally progressive LBLs after irradiation by MDA criteria, which are most relevant for early identification to prohibit delay of adequate treatment. ROC analysis was performed with the predictors “absolute attenuation on conventional/VNCa CT” and the binary outcome “locally progressive *vs*. stable/responsive lesions.”

### Analysis of Dose-Dependent Radiation Effects

To investigate a possible dose dependency of post-irradiation attenuation of LBLs, total radiation dose was correlated by Pearson’s method with the attenuation of LBLs after RT. According to the most recent ILROG guidelines, it can take up to 8 months after irradiation to discriminate persistent disease and therapy response of plasma cellular bone lesions on the gold standard imaging modalities PET/CT and MRI (2). Hence, to assure assessment of maximum therapy effects, we performed the correlation for the follow-up measurements >8 months after RT (n=106 follow-up measurements) only.

### Statistical Assessment

Statistical assessment was performed in *R* language for statistical computing, *R* Foundation, Vienna, Austria, version 4.0.0. Visualization of the data was achieved by the *R* library *ggplot2*, elegant graphics for data analysis (19).

Wilcoxon test for comparison of interval scaled data and Pearson’s linear correlation were performed in *R*. ROC analysis was used to assess the performance of attenuation measurements to discriminate irradiated *vs*. non-irradiated lesions, using the *R* library *pROC* (20). Best thresholds of the ROC analysis were determined by Youden’s method. AUCs of conventional and VNCa measurements were compared using the two-sided DeLong’s test. Statistical significance was defined as p<0.05. Data are stated as mean ± standard deviation or as median [interquartile range (IQR)]. The area under the curve (AUC) is reported with the 95% confidence interval.

Inter-rater reliability of continuous data (ROI attenuation measurements) was reported by the intraclass correlation coefficient (ICC) in a single rater type, two-way random-effects model (ICC2), using the *R* library *irr* (21, 22). Inter-rater reliability of ordinal data (tumor response by MDA criteria) was reported by *Fleiss’* Kappa coefficient using the same *R* library *irr.*

## Results

### Patients

**Figure 2:**
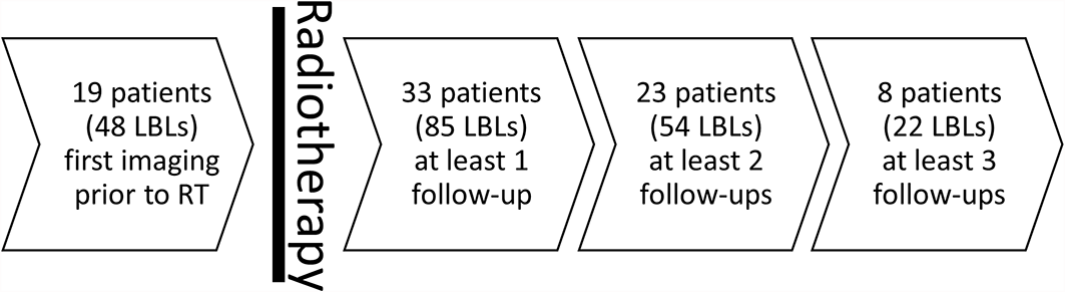
Timeline of imaging and radiotherapy.

Mean patient age at first RT was 63.3 ± 10.8 years. Twenty included patients were male, while 13 were female. The 85 included irradiated LBLs and their paired non-irradiated lesions (total number of lesions n=170) were assessed in a total number of 168 follow-up examinations after RT (median number of follow-up examinations after RT per LBL =2) yielding a median follow-up time of 400 days after RT [158.5–1,028.0 days]. Additionally, 48 irradiated LBLs plus 48 non-irradiated LBLs were assessed prior to RT (**Figure 2**).

The median total radiation dose was 30 Gy [30–36 Gy]. Radiation was applied at a median of 10 sessions [10–15] with a median single dose of 3 Gy [2–3 Gy]. Thirty-one patients received specific therapy at the timepoint of the CT scans either before and after RT (n=18) or after RT (n=13), while two patients did not receive specific systemic therapy during the follow-up period.

### Treatment Response of Individual Lesions by MD Anderson Imaging Criteria and Systemic Treatment Response by IMWG Criteria

Inter-rater variability assessment of ROI measurements and MDA ratings yielded an ICC of 0.85 (“good agreement”) and a *Fleiss’* Kappa of 0.67 (“substantial agreement”), respectively.

After irradiation, 26 LBLs underwent partial response on conventional CT images. Forty-six LBLs did not show signs of therapy response nor local progression and were rated as stable disease throughout the follow-up period. We did not observe a complete response with complete sclerotic fill-in according to MDA criteria. Local progressive disease after irradiation was observed in three LBLs. For 35 of 168 post-irradiation measurements, no previous examination was available, precluding assessment of therapy response by MDA ratings. Ten LBLs were only imaged at one timepoint after RT.

Of 168 follow-up LBL measurements, 73 were performed at timepoints of progressive disease by IMWG criteria, while 95 measurements were completed on CT scans at periods of stable disease and therapy response.

### Analysis of VNCa and Conventional Attenuation in Lytic Bone Lesions With and Without Irradiation

**Figure 3:**
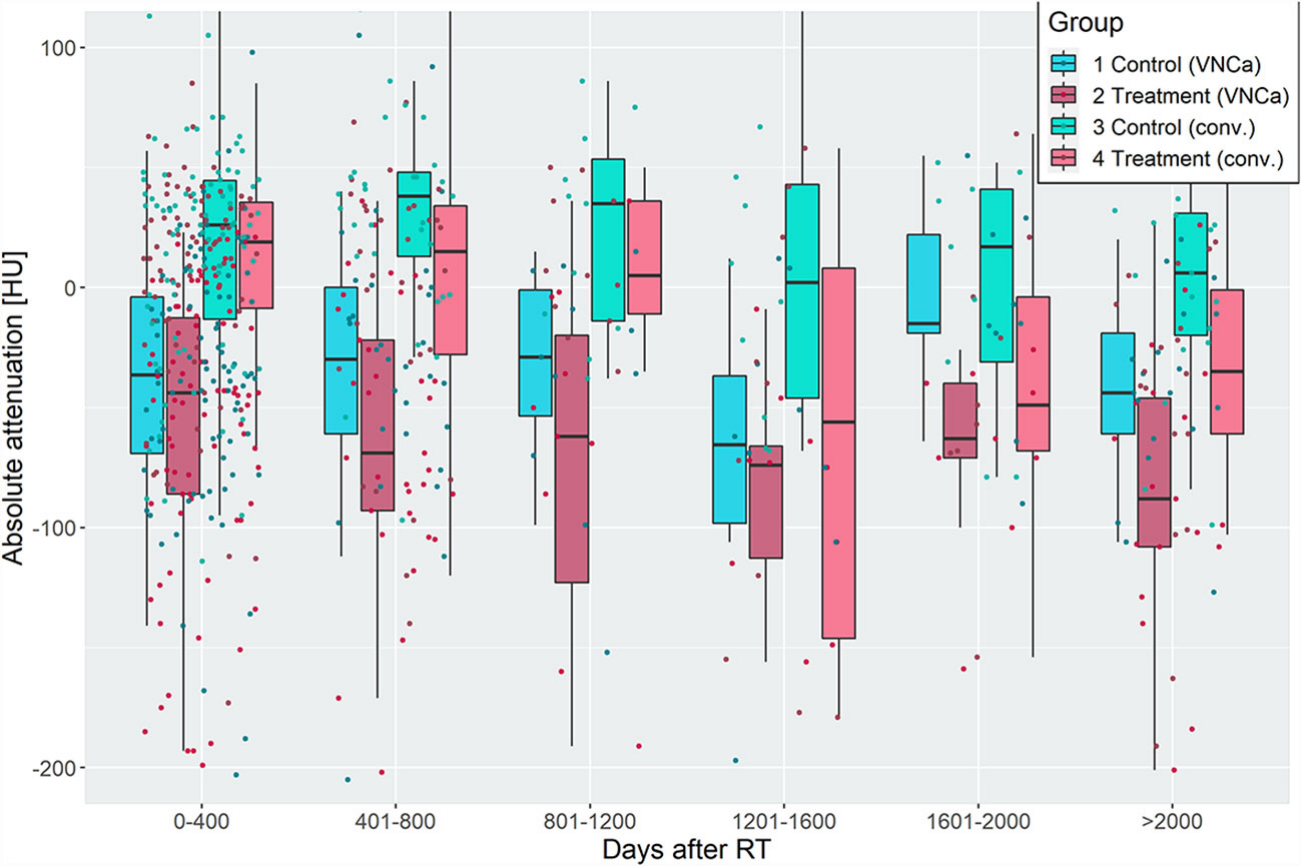
Attenuation of irradiated and non-irradiated lytic bone lesions (LBLs), grouped by time intervals of 400 days after radiotherapy. Irradiated LBLs in multiple myeloma show lower attenuation than non-irradiated lesions in the same patient. This effect is measurable on virtual noncalcium (left two boxplots of each group) and conventional (right two boxplots of each group) CT images. Discriminative performance of VNCa measurements to discriminate irradiated *vs*. non-irradiated lesions by receiver operating characteristic analysis was acceptable/excellent, except for the time intervals from 800 to 1,600 days after irradiation (see **Table 1**).

Before radiation, the median attenuation of target LBLs was 42.0 HU [30.5–47.0] and -4.5 HU [-38.0–7.0] on conventional and VNCa images, respectively. After irradiation, LBLs showed lower attenuation values than their non-irradiated, paired control lesions in the same patient. This effect varied for different time delays of imaging after irradiation. Attenuation values of irradiated and non-irradiated LBLs were grouped by their follow-up time after RT and visualized in **Figure 3**. On average, the attenuation after irradiation measured 8.5 HU [-31.8–33.0] and -53.5 HU [-94.8 to -20.5] on conventional and VNCa images, respectively.

To elaborate on the discriminative power of attenuation measurements for differentiation of irradiated *vs*. non-irradiated LBLs, a ROC analysis was performed with the predictor “absolute lesion attenuation.” The AUC for prediction of prior irradiation of a lesion varied between 0.56–0.75 for conventional and 0.57–0.85 for VNCa CT, respectively, depending on the time interval after irradiation. The AUC was generally higher for longer intervals after RT; performance of conventional and VNCa images did not significantly differ for most time intervals. The only exception was a time interval of 1,601–2,000 days after RT, when VNCa performed significantly better than conventional CT (p=0.02, two-sided DeLong’s test for paired ROC curves).

For patients with available per-irradiation DECT, we observed a change of attenuation after RT by an average of -48% [-7 to -92] on conventional and -228% [-23 to -583] on VNCa images, respectively. Respecting the percentage change of attenuation did significantly increase the AUC for VNCa images from 0.57/0.62 to 0.71/0.82 for the time intervals 0–400/401–800 days after irradiation, respectively (p<0.05, two-sided DeLong’s test for paired ROC curves). Performance of conventional CT attenuation measurements did not significantly benefit from calculating the percentage change of attenuation (p>0.05, two-sided DeLong’s test for paired ROC curves).

ROC analyses are summarized in **Table 1**.

**Table 1:** Receiver operating characteristic (ROC) analysis for differentiation of irradiated and non-irradiated bone lesions by lesion attenuation.

Days after irradiation	Conventional CT AUC	Optimum threshold	Virtual noncalcium CT AUC	Optimum threshold
0–400	0.56 [0.47–0.65]	30.5 HU	0.57 [0.49–0.66]	-40.5 HU
	attenuation change 0.68 [0.59–0.77]	-50%	attenuation change 0.71 [0.63–0.80]^#^	-117%
401–800	0.65 [0.52–0.79]	36.0 HU	0.62 [0.48–0.76]	-65.0 HU
	attenuation change 0.64 [0.56–0.72]	-50%	attenuation change 0.82 [0.69–0.96]^#^	-167%
801–1,200	0.58 [0.32–0.84]	37.0 HU	0.65 [0.40–0.89]	-43.5 HU
1,201–1,600	0.73 [0.50–0.96]	-70.0 HU	0.60 [0.33–0.87]	-63.0 HU
1,601–2,000*	0.64 [0.36–0.92]	-40.0 HU	0.85 [0.65–1.00]	-22.5 HU
>2,000	0.75 [0.59–0.91]	-7.0 HU	0.73 [0.56–0.89]	-77.0 HU

Area under the curve (AUC) is reported with 95% confidence interval, grouped by time intervals of 400 days after irradiation. For the two time intervals 0–400 and 401–800 days after irradiation, a CT scan before irradiation was available for 19 patients. For the respective bone lesions (n=48), the ROC analysis was repeated with the predictor “percentage change of attenuation before vs. after irradiation”.

*significantly higher AUC for VNCa CT compared to conventional CT (p=0.02, two-sided DeLong’s test for paired ROC curves).

^#^significantly higher AUC when performing the ROC analysis with the predictor “percentage change of attenuation before vs. after irradiation” (p<0.05, two-sided DeLong’s test for paired ROC curves).

### Subset Analysis: Lytic Bone Lesions Sorted by Their Calcium Content

**Figure 4:**
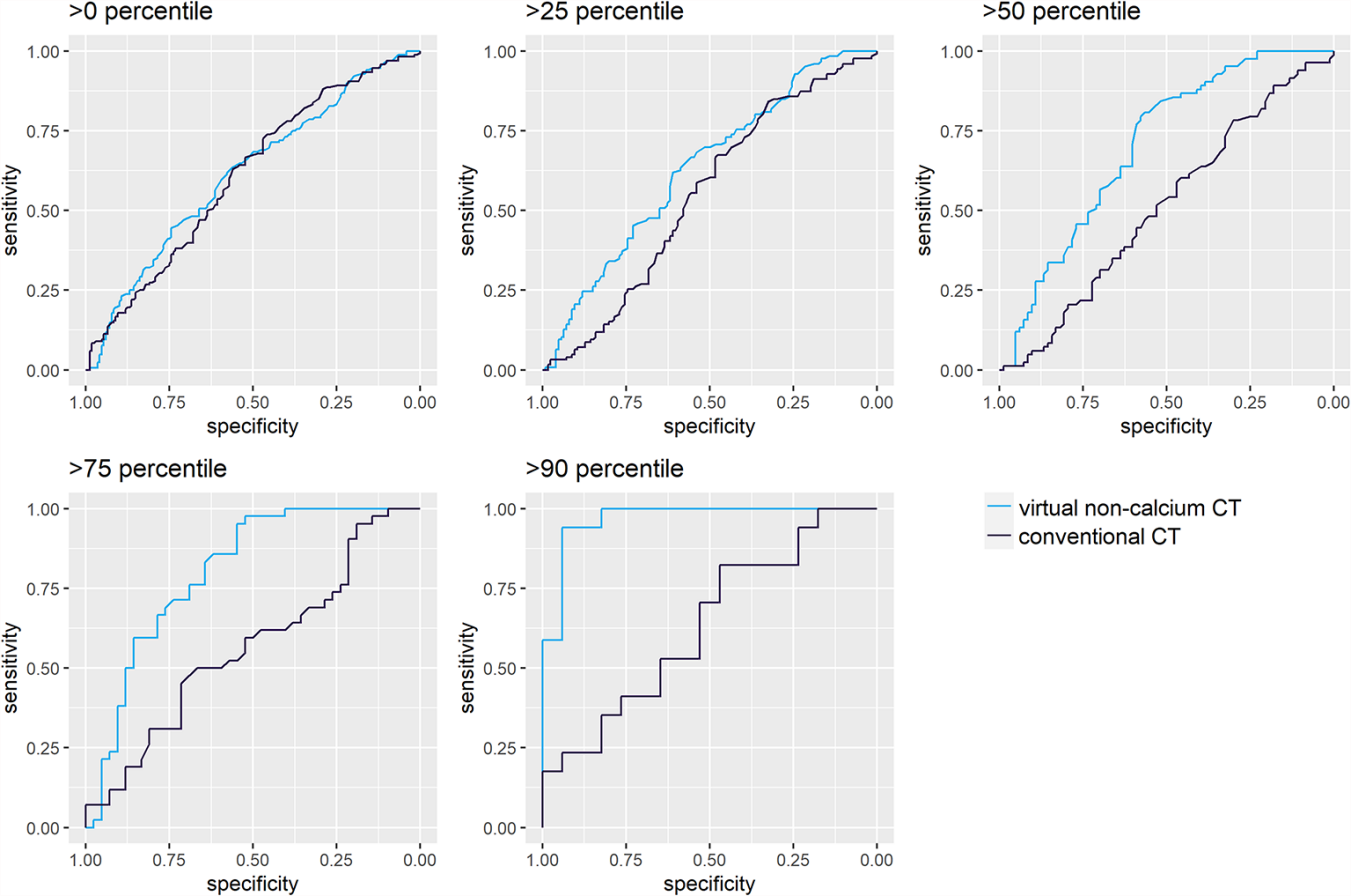
Receiver operating characteristic (ROC) analysis for differentiation of irradiated and non-irradiated lytic bone lesions, sorted by their calcium content. ROC analyses were performed for attenuation measurements on conventional (A) and virtual noncalcium (B) CT images. Darker shades of turquoise/blue represent subsets of lytic bone lesions with relatively higher calcium content. The discriminative performance of conventional CT images (A) does not vary by the calcium content of the measured lesions: The area under the curve (AUC) yields similar results between 0.51 [0.42–0.60] and 0.64 [0.45–0.83]. Conventional CT measurements could only significantly differentiate irradiated *vs*. non-irradiated lesions in the first subset of lesions >0 percentile of calcium content (Wilcoxon test, p<0.05). However, virtual noncalcium (VNCa) post-processing significantly improves the ROC’s performance, depending on the magnitude of the lesion’s calcium content: this is illustrated by the ROC curves corresponding to subsets with higher calcium content reaching further to the top left of the graph. VNCa AUC significantly exceeds the AUC of conventional CT images for the subsets with calcium content above the 25^th^ [0.63 (0.56–0.70)], 50^th^ [0.70 (0.62–0.78)], 75^th^ [0.80 (0.71–0.90)], and 90^th^ [0.96 (0.91–1.00)] percentiles (p<0.05, two-sided DeLong’s test for paired ROC curves).

LBLs with high calcium content carry the highest potential to benefit from virtual calcium suppression. Hence, the ROC analysis for differentiation of irradiated and non-irradiated lesions was repeated for four subsets of LBLs ordered by a calcium content above the 25^th^, 50^th^, 75^th^, and 90^th^ percentiles. The calcium content was estimated by a difference of *att*_conventional_ – *att*_VNCa_ *>*8.5 HU, >40 HU, >83.5 HU, and >140.5 HU, which correspond to the above-mentioned percentiles (**Figure 4**).

**Figure 5:**
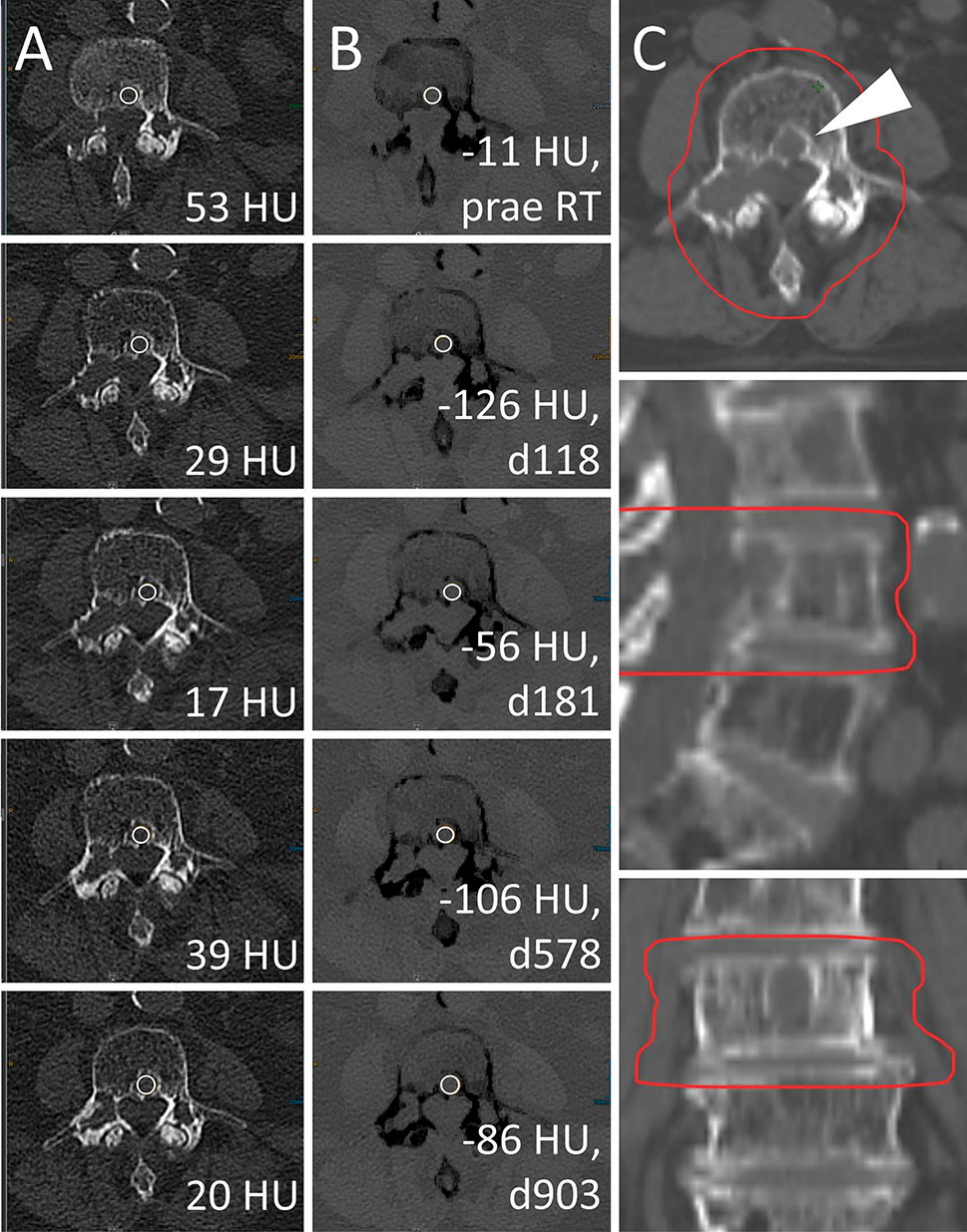
Radiotherapy (RT) response of a lytic bone lesion with high calcium content. Male, 70 years old (at time of RT) patient with history of multiple myeloma (MM). Total radiation dose was 30 Gy (3 x 10 Gy); RT planning CT is shown in column **(C)**. Axial CT slices as conventional images [column **(A)**] and virtual noncalcium (VNCa) post-processed images [column **(B)**] before RT (first row) and 118, 181, 578, and 903 days after RT (second to fifth row, respectively). On VNCa images, irradiation response in the assessed target lesion (white arrowhead) is apparent by decreasing, fatty attenuation values. Bone mineral content is estimated by subtraction of conventional and VNCa attenuation to assess lesion calcification as another sign of therapy response. In this case, the calcium specific attenuation portion increases from 64 HU prior to RT (*att*_conventional_ = 53 HU*, att*_VNCa_ = -11 HU) to a maximum of 155 HU at day 118 post-RT (*att*_conventional_ = 29 HU*, att*_VNCa_ = -126 HU), which corresponds to the 92% percentile. Attenuation measurements on conventional CT are relatively stable between 17 and 53 HU, demonstrating their poor performance to identify RT response throughout lesions with high calcium content.

For conventional CT images, differentiation of irradiated *vs*. non-irradiated bone lesions by ROC analysis was similar throughout the subsets with different calcium content (AUC 0.51 [0.42–0.60] to 0.64 [0.45–0.83]). VNCa images performed significantly better throughout subsets with higher calcium content, yielding the best AUC for the subset of lesions above the 90^th^ percentile of calcium content (AUC 0.96 [0.91–1.00], p<0.001, two-sided DeLong’s test for paired ROC curves). An exemplary lesion with higher calcium content is illustrated in **Figure 5**.

### Subset Analysis: Locally Progressive Lytic Bone Lesions After Irradiation

**Figure 6:**
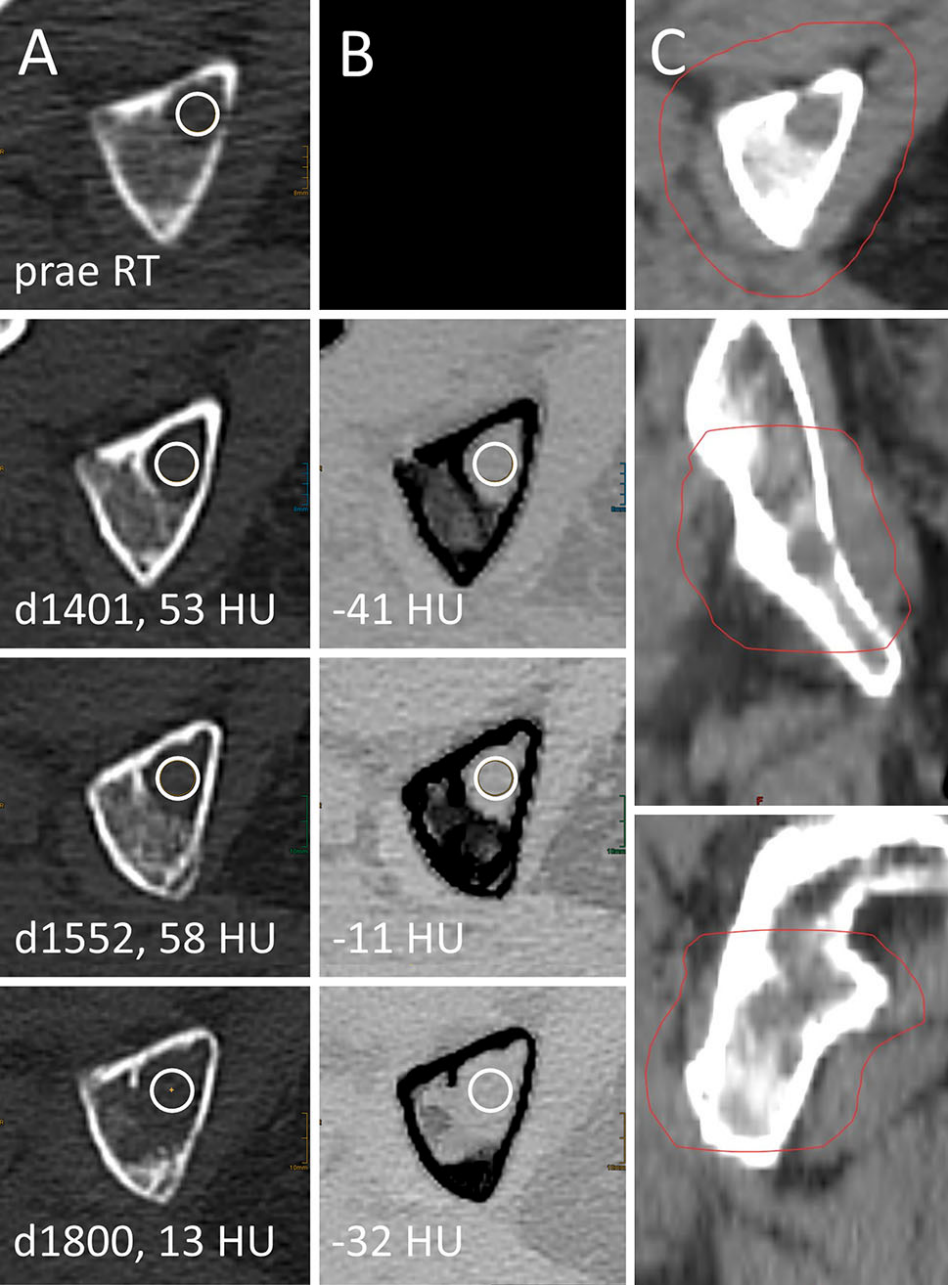
Local failure after palliative radiotherapy (RT) of a lytic bone lesion in multiple myeloma (MM). Female, 75 years old (at time of RT) patient with history of MM. Total radiation dose at the right *ischium* was 36 Gy (3 x 12 Gy); RT planning CT is shown in column **(C)**. Axial CT slices as conventional images [column **(A)**] and virtual noncalcium (VNCa) post-processed images [column **(B)**] before RT (first row) and 1,401, 1,552, and 1,800 days after RT (second to fourth row, respectively). Imaging before RT was performed on an older scanner generation, which did not match inclusion criteria to our study and hence is only shown for illustration purpose. Attenuation measurements were obtained by circular ROIs (white circles). Prior to local progression, attenuation of the LBL locates above the thresholds of 20.5 HU on conventional CT (d1401, d1552) and -27 HU on VNCa CT (d1552).

Local disease progression after RT was observed in 3 out of 75 irradiated LBLs with available previous examination for comparison. Two of these LBLs underwent delayed relapse 590 and 1,800 days after RT (**Figure 6**). Throughout follow-up examinations of LBLs, which were locally progressive at any time (n=6), attenuation values were significantly higher than attenuation of stable and responsive lesions after irradiation on conventional and VNCa images (median 32.5 HU [IQR 29.0–39.8] *vs*. 7.0 HU [IQR -35.0–29.3] on conventional images and -3.0 HU [IQR -21.8–8.3] *vs*. -62.5 HU [IQR -99.8 to -30.3] on VNCa images, respectively, one-sided Wilcoxon test, p<0.05). Best attenuation thresholds for identification of local irradiation failure in ROC analysis were >20.5 HU on conventional CT (sensitivity 1.00, specificity 0.64, AUC 0.78 [0.68–0.88]) and >-27 HU on VNCa CT (sensitivity 0.83, specificity 0.74, AUC 0.83 [0.70–0.96]).

### Attenuation of Non-Irradiated Lesions During Stable Disease and Therapy Response *vs*. Progressive Disease

Median attenuation of non-irradiated control lesions was 5.0 HU [IQR -42.5–31.5] on conventional CT and -69.0 HU [IQR -105.5 to -34.0] on VNCa CT at periods of stable disease and therapy response (n=95 follow-up measurements). During periods of progressive disease, median attenuation of control lesions was 18.0 HU [IQR -25.0–33.0] on conventional CT and -41 HU [IQR -73.0 to -9.0] on VNCa CT (n=73 follow-up measurements). On VNCa CT, control lesions demonstrated significantly higher attenuation at timepoints of progressive disease (Wilcoxon test, p<0.001); on conventional CT, this difference was not significant. Most of our findings were reproducible after splitting our dataset by systemic disease progression as an arbitrary covariate. Furthermore, our method was robust against the impact of outliers (**Supplementary Material 1**).

### Dose-Dependent Analysis of Radiation Therapy Effects

Pearson’s correlation was performed for follow-up examinations >8 months after RT. Correlation of total applied radiation dose per lesion in Gy and lesion attenuation on conventional CT in HU yielded a Pearson’s r=-0.40 [95 CI -0.55 to -0.23], slope=-4.5, p<0.001. An analogous correlation of radiation dose and lesion attenuation on VNCa images yielded a Pearson’s r=-0.21 [95 CI -0.38 to -0.02], slope=-2.2, p=0.03.

## Discussion

Since survival of MM patients is continuously increasing and side effects of radiotherapy become of rising importance, investigation of minimal effective radiation doses is at the focus of recent research (4). Regular imaging of RT response might help to address the issue of over-irradiation, based on monitoring individual lesions. Our study suggests that RT response might be assessable on standard myeloma scans in DECT. We found a decrease in attenuation and lower absolute attenuation values of irradiated LBLs after RT compared to non-irradiated lesions, with a dependency of radiation dose and the time that had passed after RT. This effect was measurable on conventional as well as VNCa post-processed images. VNCa post-processing, however, introduced a significant benefit to identify irradiated LBLs with higher calcium content (e.g., LBLs with calcium content >90^th^ percentile: AUC 0.96 [0.91–1.00] *vs*. 0.64 [0.45–0.83] for VNCa and conventional images, respectively). Calculating the percentage attenuation change of an LBL before *vs*. after RT further significantly improved discriminative performance of our VNCa measurements for the time intervals closest to the time point of irradiation (e.g. LBLs 401–800 days after RT: AUC 0.62 [0.48–0.76] *vs*. 0.82 [0.69–0.96] for absolute and relative percentage attenuation). Since fatty tissue demonstrates relatively low attenuation in CT, we hypothesize that this finding of decreasing lesion attenuation after RT correlates to fatty regression of an LBL after RT, which is an established sign of therapy response in MRI (8). Furthermore, decreasing attenuation might reflect lower cellularity of a lesion, analogously to an increasing apparent diffusion coefficient on MRI (23). VNCa measurements could only significantly discriminate irradiated *vs*. non-irradiated lesions for time intervals starting at least 6 weeks after irradiation, which might be delayed compared to diffusion weighted imaging in MRI (**Supplementary Data 2**) (23, 24).

In the above introduced three-compartment model of bone attenuation, a rise of the fatty attenuating portion (negative HU values) can be outweighed by an increase in bone mineral, calcium-like attenuation (positive HU values), which both typically constitute LBL therapy response (10). Since the calcified lesion borders were excluded during ROI placement in our study, fatty transformation and diminishing cellularity dominated over lesion sclerosis and dictated an overall lower attenuation of LBLs after irradiation. The advantage of VNCa apparently results from suppression of morphologically nonperceivable mineralization of an LBL after RT, which impedes assessment of the underlying malignant soft tissue tumor. This hypothesis is supported by the exceptional performance of VNCa measurements compared to conventional CT when analyzing LBLs with high calcium content in our study. Furthermore, this finding is in line with the recent hypothesis that the attenuation of an LBL in VNCa corresponds to its cellularity and metabolic activity (13, 25).

The suggested VNCa method can be applied to DECT data as a by-product of guideline compliant imaging, without further economic cost or radiation exposure. Several recent studies attest outstanding capabilities of VNCa imaging for diagnosis and vitality assessment of MM bone disease (5, 10, 11, 13). Evaluation of therapy response by DECT in MM is yet a novel approach of our study. Besides promising results in the context of MM, VNCa technique has recently been suggested to be feasible to assess BM pathologies such as BM edema, which traditionally is a domain of MRI (26, 27). Also for detection of metastatic spine disease, additional VNCa post-processing of DECT data was beneficial when compared to the gold standard MRI (12).

Recent research proposed a cutoff at -46.9 HU in VNCa images for differentiation of lytic bone lesions with increased metabolic activity in MM. As expected, the attenuation of LBLs prior to RT located above this threshold in our study (VNCa median -4.5 HU [-38.0–7.0]) since metabolically active LBLs might more likely qualify for irradiation (13). The best attenuation threshold for identification of local radiation failure in our study was >-27.0 HU on VNCa CT (AUC 0.83, sensitivity 0.83, specificity 0.74). We hypothesize that the drop of attenuation below threshold for identification of infiltrated BM might indicate therapy response. On the other hand, an exceptionally high VNCa attenuation after RT might allow for early recognition of rarely occurring local radiation failure, even before progression of osteolysis.

Our study has several limitations, which need to be discussed. First, discrimination of irradiated and non-irradiated bone lesions between 800 and 1,600 days after RT was somehow poor and resulted in large confidence intervals, possibly due to the small number of patients in this group. Furthermore, we did not include gold standard imaging from a second modality such as MRI or clinical therapy response as validation for our findings. The relationship of radiological response and clinical response in MM is a common limitation for investigation of novel imaging approaches, which has evoked dedicated studies by itself (28, 29). Such investigation should also follow for external validation of our findings and correlation with clinical outcomes but was beyond the scope of our feasibility study. Still, we found evidence that argues for a relationship between our measurements and local therapy response: First, attenuation of an MM lesion in VNCa is considered to estimate its cellularity and metabolic activity. Lower attenuation after irradiation suggests lower cellularity and vitality, possibly indicating therapy response. *Vice versa*, local tumor progression and irradiation failure in our study were preceded by exceptionally high VNCa attenuation of a lesion. Secondly, concerning non-irradiated lesions, our findings resembled the clinical gold standard of IMWG response criteria as expected: non-irradiated lesions demonstrated significantly higher VNCa attenuation during periods of systemic disease progression. Lastly, clinical therapy response after irradiation is dose dependent (30). Analogously, we found a dose-dependent decrease in attenuation after irradiation. Our analysis was further limited by the fact that VNCa images were only available for 19 out of 33 patients before RT, which partially precluded calculation of percentage attenuation changes after RT. Furthermore, inconsistent regimes of systemic therapy throughout our study population and different genotypes of MM with varying malignancy might bias our data. However, our study design aims to minimalize such bias, since for every irradiated lesion, a non-irradiated control lesion was analyzed. This should prevent systemic confounders other than our main investigated independent variable “application of RT” from affecting our measurements, which, to the best of our knowledge, is a novel approach. Furthermore, conclusions about RT failure are delimitated by rare occurrence of local disease progression. Lastly, our study is limited by its retrospective characteristics.

Future studies should focus on investigation of robust cutoffs for therapy response assessment of MM bone disease. However, multicenter and multivendor studies are needed for validation of absolute cutoff values. Automated, AI-supported segmentation might help to address biases introduced by human readers.

In conclusion, our findings suggest that lesion-by-lesion irradiation response might be assessable on standard myeloma scans in DECT. This might permit lesion-specific optimization of radiation regimens for minimal RT side effects and allow for early recognition of local radiation failure.

## Data Availability Statement

The raw data supporting the conclusions of this article will be made available by the authors, without undue reservation.

## Ethics Statement

The studies involving human participants were reviewed and approved by the University of Cologne, Faculty of Medicine, Institutional Review Board. Written informed consent for participation was not required for this study in accordance with the national legislation and the institutional requirements.

## Author Contributions

PF, NG, and CB contributed to conception and design of the study. SR and PF built the patient population. GB, EC, JK, and PF performed the investigation and data collection. PP-S and JK performed the statistical analysis. PF wrote the first draft of the manuscript. SL, NG, CB, and DM wrote sections of the manuscript. DM and NG supervised the project. All authors contributed to manuscript revision, read, and approved the submitted version.

## Conflict of Interest

NH receives research support from Philips Healthcare outside the submitted work. NH and DM are on the speaker’s bureau of Philips Healthcare.

The remaining authors declare that the research was conducted in the absence of any commercial or financial relationships that could be construed as a potential conflict of interest.

## Publisher’s Note

All claims expressed in this article are solely those of the authors and do not necessarily represent those of their affiliated organizations, or those of the publisher, the editors and the reviewers. Any product that may be evaluated in this article, or claim that may be made by its manufacturer, is not guaranteed or endorsed by the publisher.
