# Supplementary material for: Radiotherapy Response Assessment of Multiple Myeloma: A Dual-Energy CT Approach With Virtual Non-Calcium Images
Source: Front Oncol. 2021 Sep 23;11:734819. doi: 10.3389/fonc.2021.734819 (PMC8504158; doi:10.3389/fonc.2021.734819)
Supplement: Supplementary file 1 [file DataSheet_1.docx]

Supplementary Material 1: Analysis of statistical robustness.

**Assessment of robustness against outliers.**

To ensure that our findings are not induced by outliers, we minimized the influence of outliers by transforming all HU measurements to a logarithmic scale, followed by a repetition of our analyses. Before performing the logarithmic transformation, HU measurements were shifted to positive values >0 HU in a linear fashion. The analogous plot to Figure 3 of the main manuscript is demonstrated in Supplementary Figure 1.


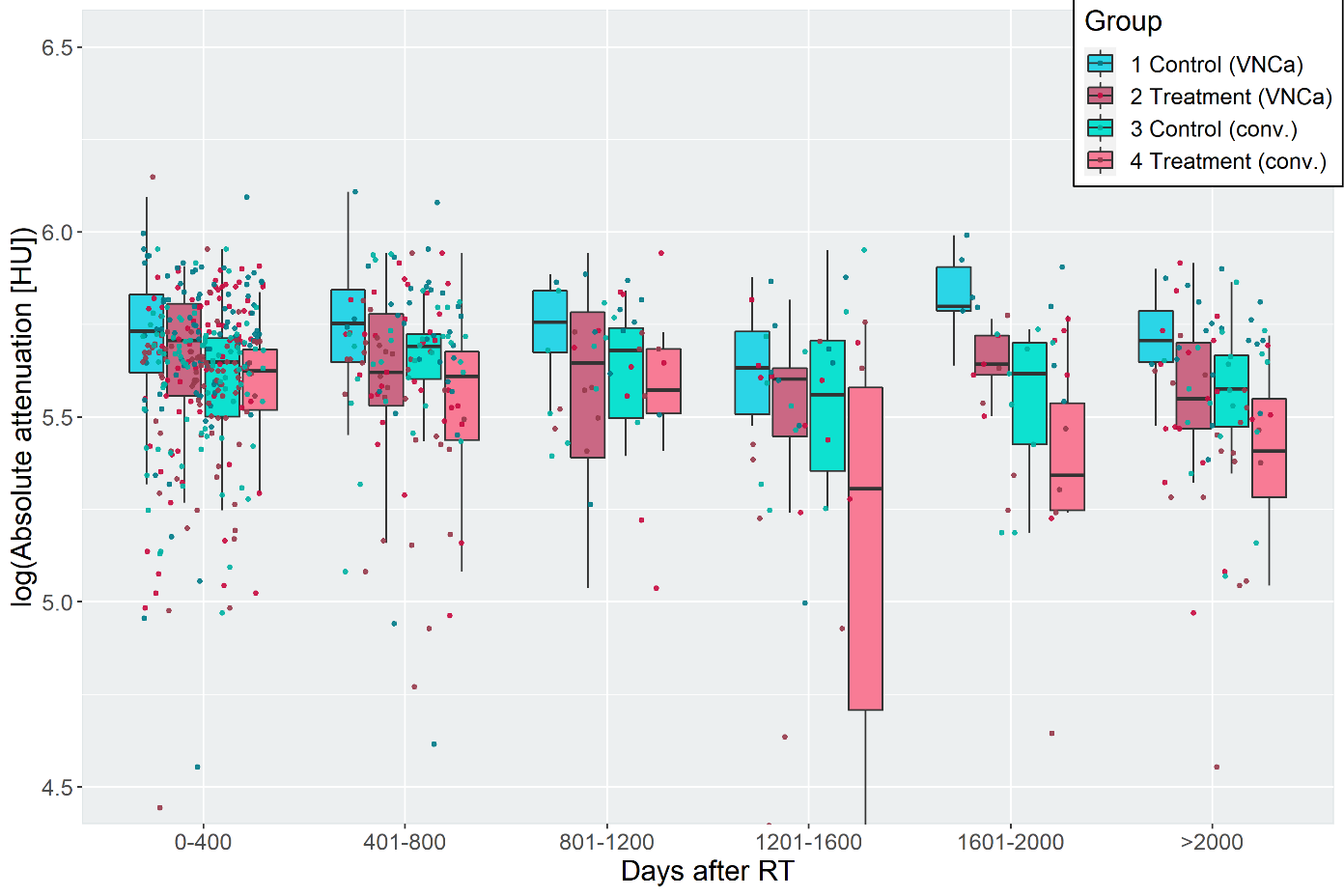


Supplementary Figure 1. Attenuation of irradiated and non-irradiated lytic bone lesions (LBLs) on a **logarithmic scale**, grouped by time intervals of 400 days after radiotherapy.

Attenuation measurements were transformed to a logarithmic scale, in order to investigate robustness of our methodology against outliers. Analogously to Figure 3 of the main manuscript, irradiated LBLs in multiple myeloma show lower attenuation than non-irradiated lesions in the same patient. This effect is measurable on virtual non-calcium (two left boxplots of each group) and conventional (two right boxplots of each group) and CT images.

On the logarithmic scale, the change of attenuation before vs. after irradiation was calculated as the difference of attenuation, rather than percentage change. Distribution of percentiles and receiver operating characteristic (ROC) curves are not affected by the transformation, because the numerical order of values does not differ between logarithmic and linear scales. Hence, Wilcoxon tests were performed analogously to ROC analyses of the main document, first grouped by time intervals after radiotherapy (analogously to Table 1) and secondly their calcium content (analogously to Figure 4):

1. Conventional HU measurements on a logarithmic scale could significantly differentiate irradiated vs. non-irradiated lesions in the time intervals from 400-800 days and >2000 days after radiotherapy (Wilcoxon test, p<0.05). Virtual non-calcium (VNCa) HU measurements on a logarithmic scale achieved a significant differentiation in the time intervals 0-400 days, 1600-2000 days, and >2000 days after radiotherapy (Wilcoxon test, p<0.05). Considering the change of attenuation before vs. after irradiation, discrimination of irradiated and non-irradiated lesions was significant for 0-400 days, and 0-400 as well as 400-800 days after irradiation for conventional and VNCa images, respectively. The time intervals with significantly different Wilcoxon tests mostly correspond to time intervals with acceptable/excellent performance of ROC analysis in the main manuscript.
2. VNCa HU measurements on a logarithmic scale significantly differentiated irradiated vs. non-irradiated lesions ordered by their calcium content throughout all subsets (above the 25^th^, 50^th^, 75^th^, and 90^th^ percentiles, Wilcoxon test, p<0.05). Conventional HU measurements were only significantly different without selection by calcium content (>0 percentile, Wilcoxon test, p<0.05). This finding resembles the results of the respective analysis in the main manuscript.

Discriminative power of conventional and VNCa HU measurements was robust after minimizing the influence of outliers by applying a logarithmic transformation.

**Assessment of robustness against systemic disease progression or therapy response.**

Systemic disease progression or therapy response might have an impact on therapy response assessment of individual, irradiated lesions. E. g., at timepoints of systemic therapy response, the systemic effects to an individual lesion might be falsely attributed to a local irradiation effect. To ensure robustness of our findings against systemic effects, we split our dataset by progressive (n=73 measurements) and therapy response/stable (n=95 measurements) disease by IMWG criteria at the timepoint of imaging, followed by an individual analysis of both subsets.

Results for progressive and therapy response/stable lesions are presented individually in Supplementary Figure 2 and 3, as well as Supplementary Table 2 and 3, analogously to Figure 3, 4, and Table 1 of the main manuscript.


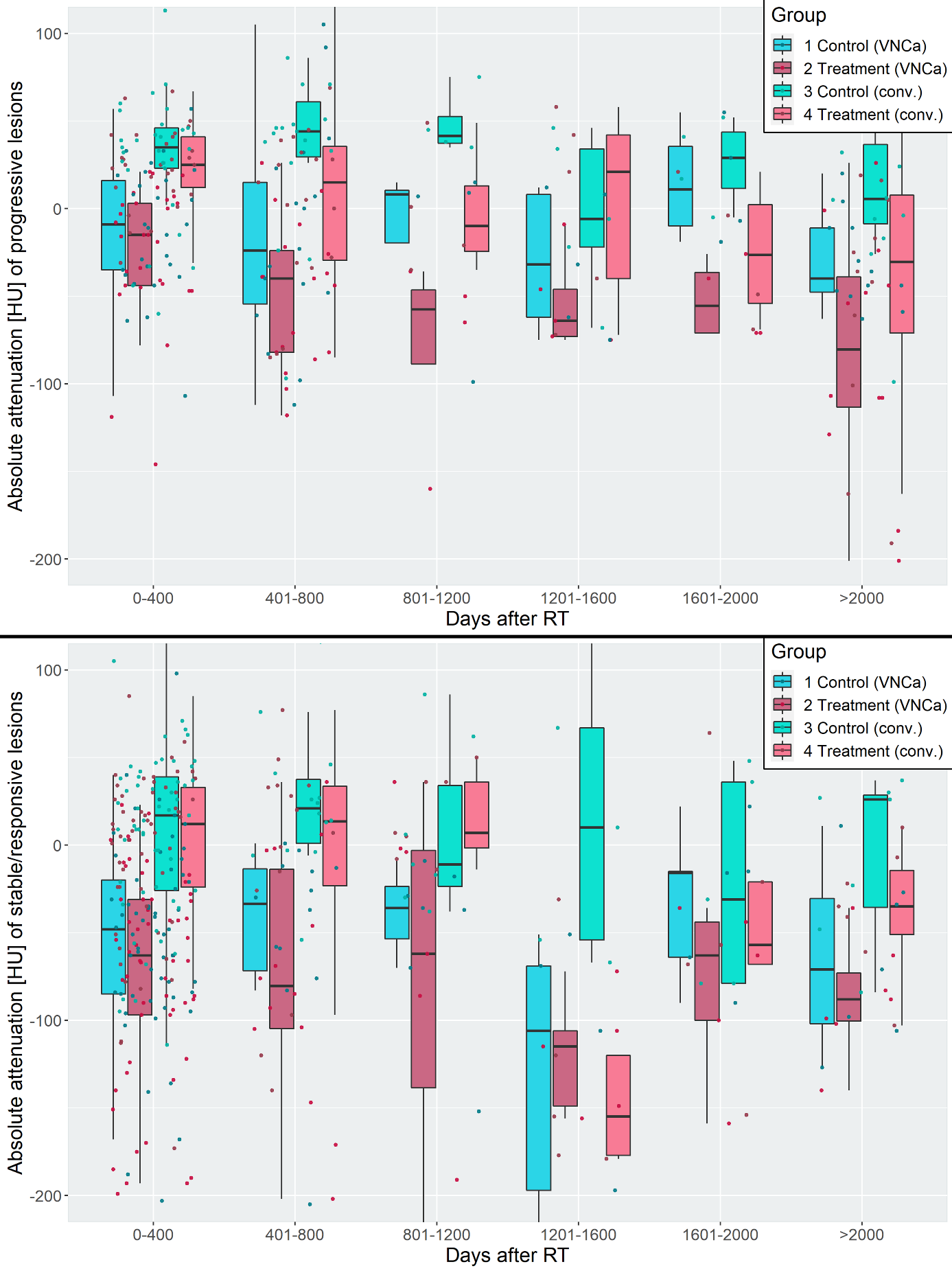


Supplementary Figure 2. Attenuation of irradiated and non-irradiated lytic bone lesions (LBLs), **grouped by IMWG response criteria** at the timepoint of imaging

Top row: Subset of lesions with progressive disease at the timepoint of imaging.
Bottom row: Subset of lesions with stable/responsive disease at the timepoint of imaging.

Supplementary Table 2. Receiver operating characteristic (ROC) analysis for differentiation of irradiated and non-irradiated bone lesions at timepoints of **progressive disease**.

| Days after irradiation | Conventional CT AUC | Optimum threshold | Virtual non-calcium CT AUC | Optimum threshold |
| --- | --- | --- | --- | --- |
| 0-400 | 0.60 [0.45 – 0.75] | 30.5 HU | 0.60 [0.45 – 0.75] | 12.5 HU |
|  | attenuation change  0.67 [0.49 – 0.86] | -6% | attenuation change  0.67 [0.48 – 0.87] | +2% |
| 401-800 | 0.71 [0.53 – 0.88] | 32.5 HU | 0.62 [0.44 – 0.81] | -25.0 HU |
|  | attenuation change  0.55 [0.28 – 0.82] | -1% | attenuation change  0.85 [0.68 – 1.00] | -256% |
| 801-1200 | 0.81 [0.43 – 1.00] | 18.0 HU | 0.81 [0.43 – 1.00] | -14.5 HU |
| 1201-1600 | 0.52 [0.11 – 0.93] | 38.0 HU | 0.70 [0.33 – 1.00] | -39.0 HU |
| 1601-2000 | 0.81 [0.48 – 1.00] | 6.5 HU | 1.00 [NA – 1.00] | -22.5 HU |
| >2000 | 0.75 [0.55 – 0.95] | -21.0 HU | 0.72 [0.50 – 0.94] | -85.0 HU |

Area under the curve (AUC) is reported with 95% confidence interval, grouped by time intervals of 400 days after irradiation.

Supplementary Table 3. Receiver operating characteristic (ROC) analysis for differentiation of irradiated and non-irradiated bone lesions at timepoints of **therapy response/stable disease**.

| Days after irradiation | Conventional CT AUC | Optimum threshold | Virtual non-calcium CT AUC | Optimum threshold |
| --- | --- | --- | --- | --- |
| 0-400 | 0.55 [0.44 – 0.65] | 43.5 HU | 0.57 [0.47 – 0.68] | -41.5 HU |
|  | attenuation change  0.69 [0.59 – 0.79] | +7% | attenuation change  0.75 [0.66 – 0.84] | -137% |
| 401-800 | 0.58 [0.36 – 0.81] | -10.5 HU | 0.59 [0.35 – 0.82] | -84.0 HU |
|  | attenuation change  0.73 [0.48 – 0.97] | -83% | attenuation change  0.78 [0.54 – 1.00] | -167% |
| 801-1200 | 0.63 [0.28 – 0.98] | -15.5 HU | 0.51 [0.14 – 0.88] | -78.0 HU |
| 1201-1600 | 0.92 [0.74 – 1.00] | -93.5 HU | 0.54 [0.09 – 0.99] | -70.5 HU |
| 1601-2000 | 0.52 [0.11 – 0.93] | 7.5 HU | 0.76 [0.41 – 1.00] | -26.0 HU |
| >2000 | 0.71 [0.41 – 1.00] | 18.0 HU | 0.63 [0.30 – 0.96] | -35.0 HU |

Area under the curve (AUC) is reported with 95% confidence interval, grouped by time intervals of 400 days after irradiation.


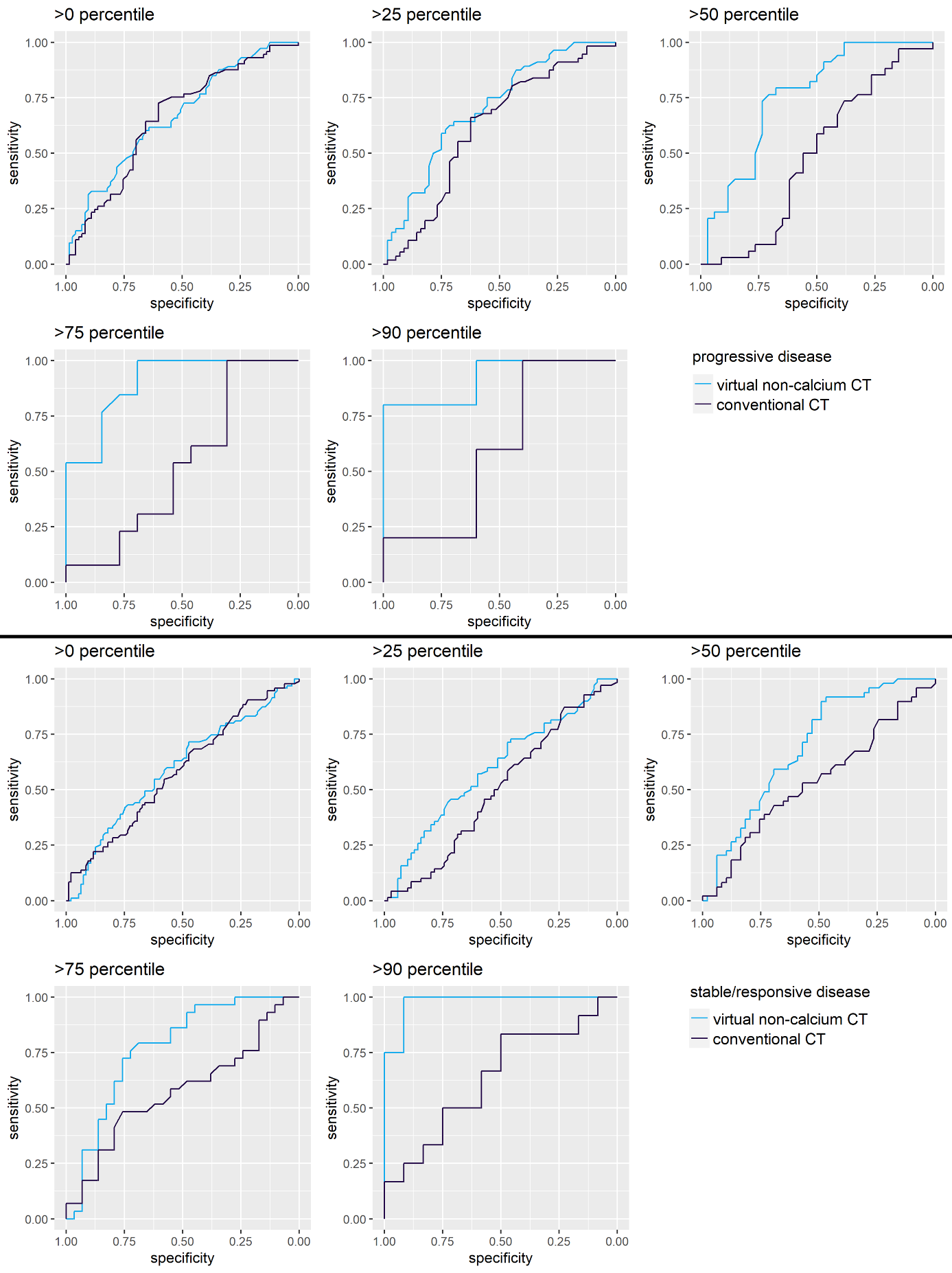


Supplementary Figure 3. Receiver operating characteristic (ROC) analysis for differentiation of irradiated and non-irradiated lytic bone lesions, sorted by their calcium content and **grouped by IMWG response criteria.**

Top row: Subset of lesions with progressive disease at the timepoint of imaging. AUC ranged from 0.49 [0.34 – 0.63] to 0.66 [0.57 – 0.75] for conventional CT measurements and from 0.67 [0.58 – 0.75] to 0.92 [0.74 – 1.00] for VNCa measurements, respectively. VNCa was significantly superior to conventional measurements for lesions with calcium content above the 50^th^ and 75^th^ percentile (p<0.05, two-sided DeLong’s test for paired ROC curves).
Bottom row: Subset of lesions with stable/responsive disease at the timepoint of imaging. AUC ranged from 0.50 [0.40 – 0.60] to 0.64 [0.41 – 0.87] for conventional CT measurements and from 0.59 [0.51 – 0.68] to 0.98 [0.93 – 1.00] for VNCa measurements, respectively. VNCa was significantly superior to conventional measurements for lesions with calcium content above the 75^th^ and 90^th^ percentile (p<0.05, two-sided DeLong’s test for paired ROC curves).

Individual results for the two subsets progressive and therapy response/stable disease are generally resembling the findings of the main document. I.e., HU measurements on VNCa and conventional CT are lower after radiotherapy compared to non-irradiated lesions, depending on the time that has passed after irradiation. At periods of therapy response/stable disease, discriminative performance at 800-1600 days after irradiation is somehow poor. The subset analysis of lesions with relative high calcium content could mostly reproduce the findings of the main manuscript for measurements during progressive and therapy response/stable disease. Here, VNCa imaging was significantly superior to conventional CT, depending on a lesion’s calcium content.

Concluding, our findings were mostly robust against splitting the dataset into measurements at periods of progressive and therapy response/stable disease. However, the advantage of VNCa vs. conventional measurements for fixed time intervals after irradiation could not be significantly reproduced, which we consider an effect of the smaller sample size after subdivision. We did not find substantial divergency to the findings of the main manuscript; hence, we consider our methodology of measuring individual lesions robust against systemic progress or therapy response of the disease. This is consistent with the concept of our study design to measure irradiated and non-irradiated lesions in the same patient at identical timepoints: Thus, systemic disease progress or therapy response is not expected to cause systematic bias by affecting only one of the compared groups. Further, our results proved robust against alternative HU measurements (e.g., logarithmic transformation); we did not find evidence that our conclusions can be attributed to the impact of outliers.
