# Supplementary material for: Radiotherapy Response Assessment of Multiple Myeloma: A Dual-Energy CT Approach With Virtual Non-Calcium Images
Source: Front Oncol. 2021 Sep 23;11:734819. doi: 10.3389/fonc.2021.734819 (PMC8504158; doi:10.3389/fonc.2021.734819)
Supplement: Supplementary file 2 [file DataSheet_2.docx]

Supplementary Material 2: Early therapy response after irradiation

Diffusion weighted imaging in magnetic resonance imaging (MRI) can assess therapy response in multiple myeloma already after a few weeks (e. g. Horger et al., three weeks after therapy) (1,2). To investigate the capabilities of virtual non-calcium (VNCa) imaging to demonstrate early therapy response, we examined the subset of patients with early follow-up dual-energy CT (DECT) after irradiation (<20 weeks, n=14 follow-up DECTs, Supplementary Figure 1).


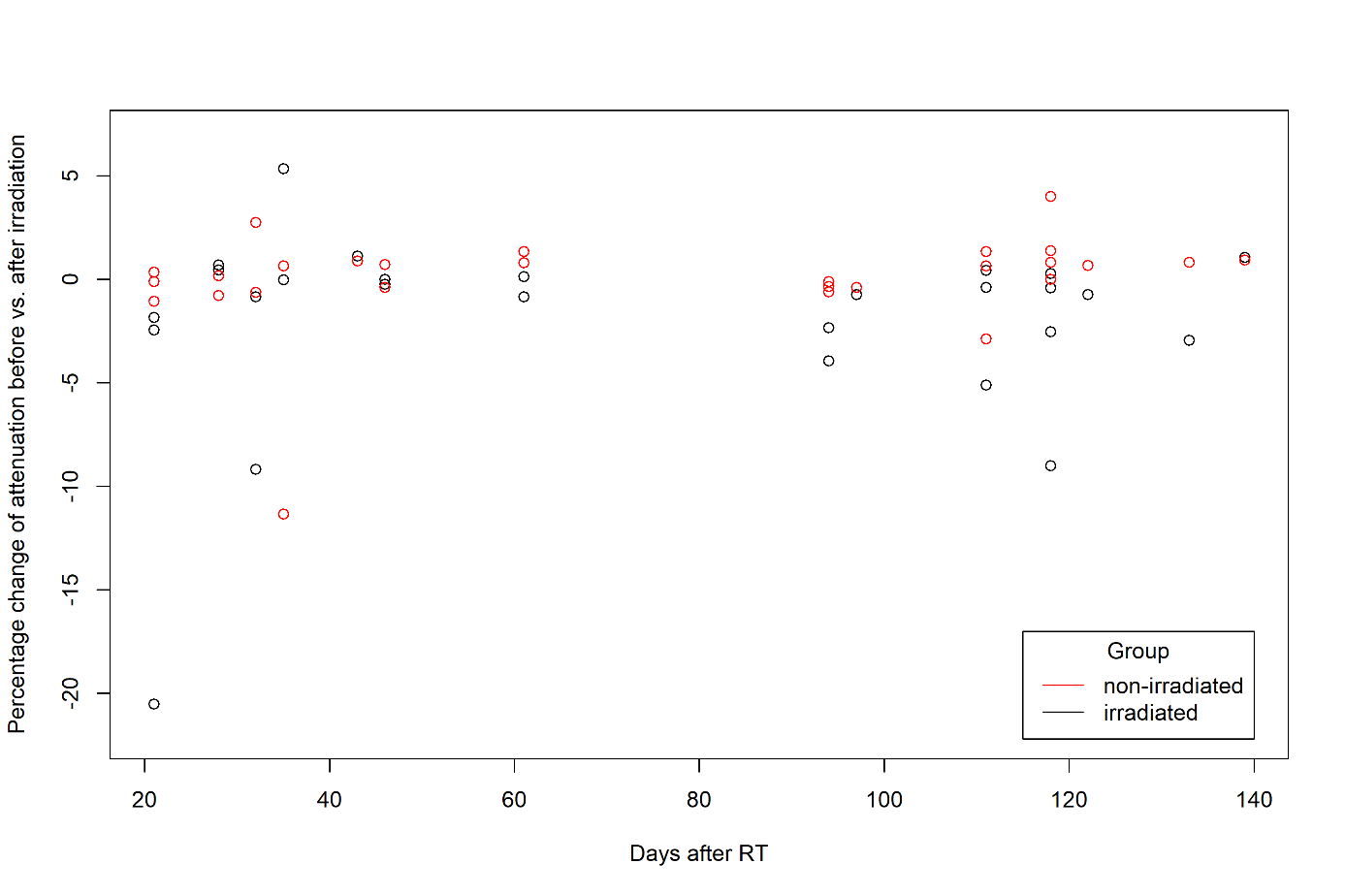


**Supplementary Figure 1: Subset analysis of patients with early (<20 weeks) follow-up dual-energy CT after irradiation.**

For early follow-up examinations between 6-20 weeks after irradiation, irradiated lesions demonstrated a stronger decrease of attenuation or a less pronounced increase of attenuation after radiotherapy, compared to non-irradiated lesions. Discriminative performance of receiver operating characteristic analysis was excellent for this period (AUC 0.80 [0.65 – 0.94], best threshold 0.5%). Yet, for very early follow-ups during the first six weeks after irradiation, this effect did not demonstrate acceptable performance within our population.

For patients with early follow-up DECT between 6-20 weeks after radiotherapy, irradiated lesions were discriminated excellently by a stronger decrease or a less pronounced increase of attenuation, compared to non-irradiated lesions (AUC 0.80 [0.65 – 0.94], best threshold 0.5%). This finding is in line with the mid- to long-term follow-ups demonstrated in the main document. Very early follow-up DECTs <6 weeks after irradiation were not adequate for discrimination of irradiated and non-irradiated lesions by VNCa measurements; however, we refrain for a final evaluation due to our small sample size (n=4 follow-up examinations / 9 irradiated lesions with follow-up <6 weeks).

**References**

1. Messiou C, Giles S, Collins D, West S, Davies F, Morgan G, Desouza N. Assessing response of myeloma bone disease with diffusion-weighted MRI. *Br J Radiol* (2012) **85**: doi:10.1259/BJR/52759767

2. Horger M, Weisel K, Horger W, Mroue A, Fenchel M, Lichy M. Whole-body diffusion-weighted MRI with apparent diffusion coefficient mapping for early response monitoring in multiple myeloma: preliminary results. *AJR Am J Roentgenol* (2011) **196**: doi:10.2214/AJR.10.5979
