# Supplementary figures and images for: Radiotherapy Response Assessment of Multiple Myeloma: A Dual-Energy CT Approach With Virtual Non-Calcium Images
Source: Front Oncol. 2021 Sep 23;11:734819. doi: 10.3389/fonc.2021.734819 (PMC8504158; doi:10.3389/fonc.2021.734819)

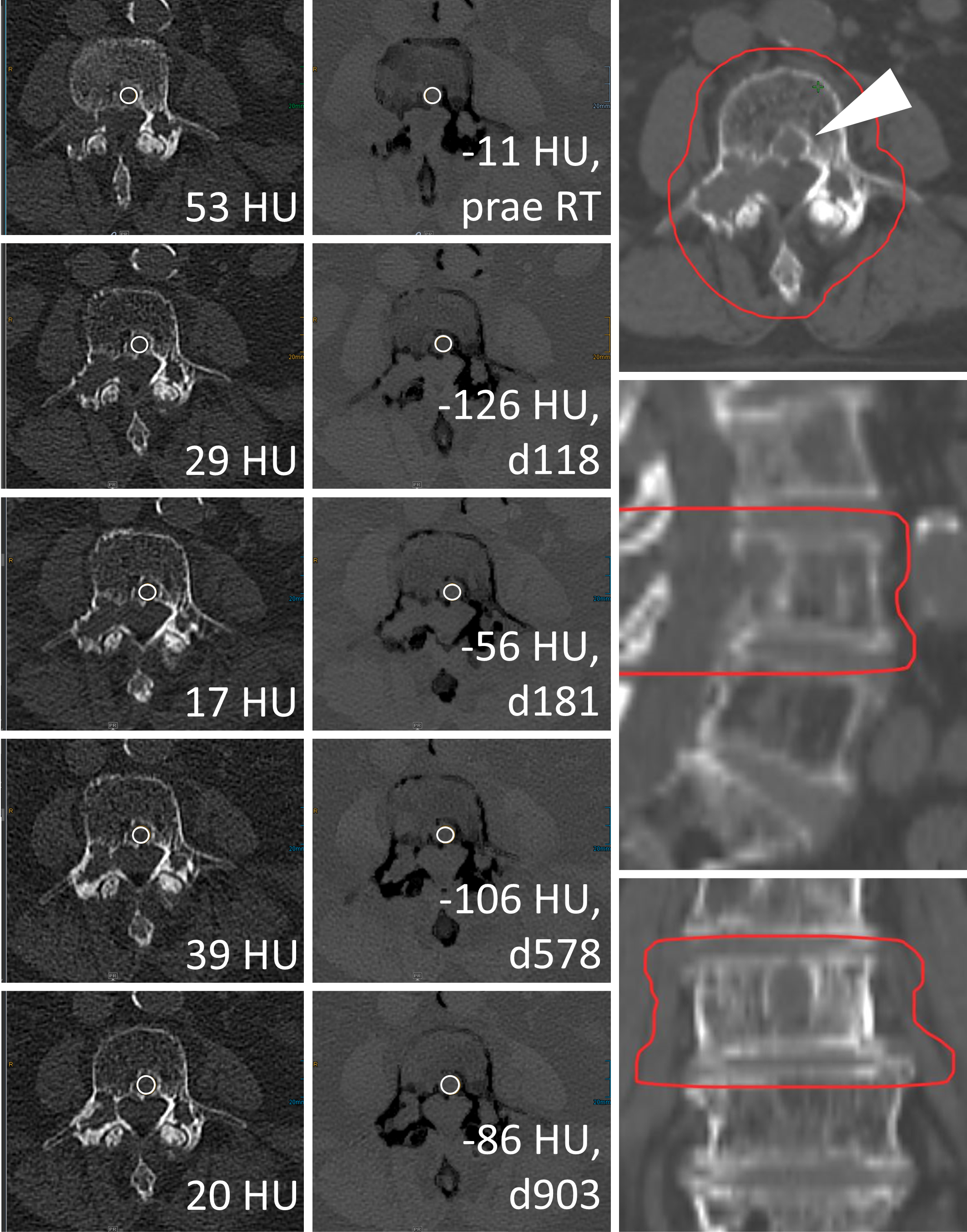

Supplement: Supplementary file 3 [file Image_1.tif]

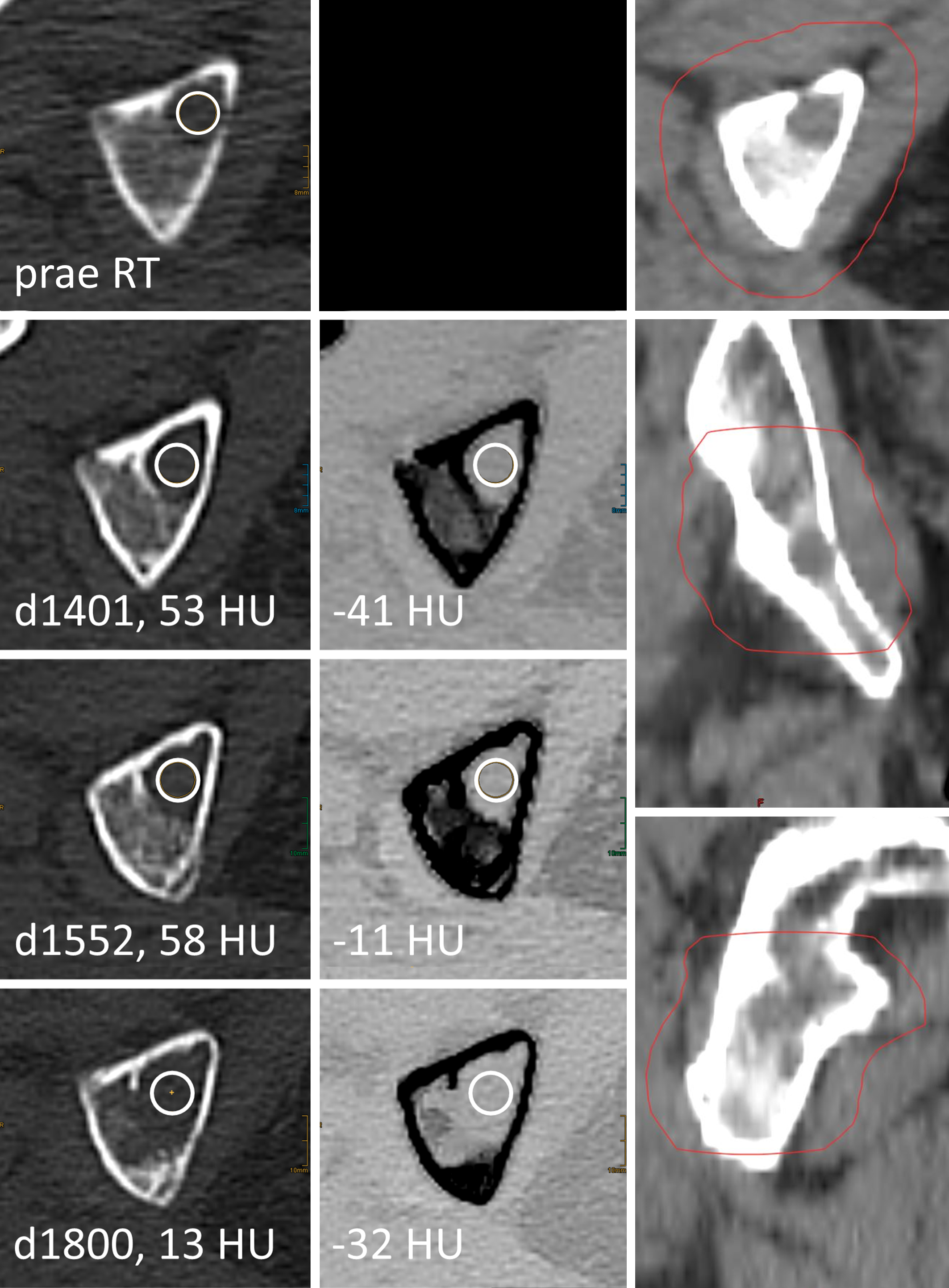

Supplement: Supplementary file 4 [file Image_2.tif]
